# Associations Between Difficulties in Emotion Regulation and Post-Traumatic Stress Disorder in Deployed Service Members of the German Armed Forces

**DOI:** 10.3389/fpsyt.2020.576553

**Published:** 2020-09-15

**Authors:** Jan Peter Spies, Jan Christopher Cwik, Gert Dieter Willmund, Christine Knaevelsrud, Sarah Schumacher, Helen Niemeyer, Sinha Engel, Annika Küster, Beate Muschalla, Kai Köhler, Deborah Weiss, Heinrich Rau

**Affiliations:** ^1^ Department of Clinical Psychology and Psychotherapy, Faculty of Human Sciences, University of Cologne, Cologne, Germany; ^2^ Department for Military Mental Health, German Armed Forces Military Hospital Berlin, Berlin, Germany; ^3^ Division of Clinical Psychological Intervention, Department of Education and Psychology, Freie Universität Berlin, Berlin, Germany; ^4^ Department of Clinical Psychology and Psychotherapy, Institute of Psychology, Technische Universität Braunschweig, Braunschweig, Germany

**Keywords:** posttraumatic stress disorder, German Armed Forces, veterans, service members, deployment, emotion regulation, moral injury, social acknowledgment

## Abstract

**Background:**

Experiencing a traumatic event can lead to post-traumatic stress disorder (PTSD), but not every traumatized person develops PTSD. Several protective and risk factors have been identified in civilians and veterans to explain why some individuals develop PTSD and others do not. However, no research has confirmed the relationship between emotion regulation and PTSD in deployed German Armed Forces service members after a foreign assignment. Previous studies have identified some protective factors, such as social support, social acknowledgment, specific personal values, and posttraumatic growth, as well as risk factors, like moral injury and emotion regulation. Thus, the aim of the present study is to confirm the relationship between emotion regulation and PTSD and to test for factors that are associated with higher severity of PTSD symptoms in such a sample.

**Methods:**

A *post-hoc* secondary analysis was conducted on data collected in a randomized controlled trial. Participants (*N* = 72) were male active and former military service members that have returned from deployment and were recruited from the German Armed Forces. These participants were separated into two groups according to PTSD diagnosis based on the results of a structured diagnostic interview. Data from evaluation questionnaires administered upon entry into the study were subjected to a cross-sectional analysis. The measures included the severity of PTSD symptoms, clusters of PTSD symptoms, clinical measures, and several measures assessing PTSD-related constructs. Analyses included the Spearman rank correlation coefficient, X^2^ tests for nominal data, Mann-Whitney U-tests for non-parametric data, and a mediation analysis.

**Results:**

The results of the mediation analysis revealed that difficulties in emotion regulation were significantly associated with the severity of PTSD symptoms, which was mediated by social acknowledgment and experimental avoidance but not by moral injury. The analyses showed that the severity of PTSD symptoms and all clusters of PTSD symptoms were significantly associated with most of the measured constructs in expectable directions. Participants in the PTSD group showed significantly higher mean scores on questionnaires measuring constructs that have been associated with PTSD, like emotion regulation and moral injury. They also showed lower mean scores in questionnaires for social support and social acknowledgment as a victim or survivor than participants in the non-PTSD group.

**Conclusion:**

The present results show that difficulties in emotion regulation are directly associated with the severity of PTSD symptoms in service members of the German Armed Forces. This association is mediated by social acknowledgment and experimental avoidance, but not by moral injury. Thus, future studies should investigate these potentially crucial factors for better understanding of the development and maintenance of PTSD in service members of the German Armed Forces after deployment to create possible treatment adaptions.

**Clinical Trial Registration:**

Australian Clinical Trials Registry, identifier ACTRN 12616000956404 http://www.anzctr.org.au/Trial/Registration/TrialReview.aspx?id=370924.

## Introduction

The diagnosis of posttraumatic stress disorder (PTSD) was firstly listed as a codable syndrome in the third version of the Diagnostic and Statistical Manual of Mental Disorders (DSM) (1). PTSD involves symptoms of re-experiencing, avoidance, and hyperarousal associated with a traumatic event. Since the introduction of the fifth edition of the DSM (DSM-5), symptoms of persisting negative cognitions and mood were added as a further cluster of symptoms (2). The symptoms of PTSD result in severe health restrictions and can seriously affect quality of life (3).

Even though experiencing a traumatic event can lead to PTSD, not every traumatized person develops PTSD (4). The lifetime prevalence of PTSD is 6.8% for civilians in the USA (5). For German civilians, the 12-month prevalence of PTSD is 2.3% (6). Compared with civilians, service members have a higher risk of developing PTSD, and deployed service members have a higher risk of developing it than undeployed service members (7).

Among deployed service members, it is possible to develop PTSD after one incident, yet there is growing evidence that various deployments or various incidents lead to a higher risk of developing it (8, 9). In general, PTSD remains a significant problem among service members after a foreign assignment (10). The prevalence rates range from 4% for British veterans to 9–20% for US veterans (11, 12). However, service members in the German Armed Forces (GAF) show lower prevalence rates, which range from 2.9% for deployed service members (10) to 3.2% for deployed service members with combat exposure (7). Yet presumably, nearly half of all GAF military personnel who suffer from PTSD after deployment are neither diagnosed nor reported (10). In the armed forces of other nations, it is also likely that the estimated number of unknown cases is higher than reported (9).

Risk factors have also been identified for the development of PTSD that do not apply exclusively to the military context. These factors comprise individual factors that are also reported in civilian samples, such as persisting psychological disorder (13, 14) or negative appraisals and cognition (15). Emotion regulation (ER) is one predictor that has repeatedly been identified as crucial for the development of PTSD (16–18). ER is defined as the deliberate or unintentional process of influencing the experience of emotions and their intensity (19). Thus, ER has to be distinguished from coping and other related constructs (20).

The profile of applied ER strategies of an individual coping with PTSD may even predict the overall symptom severity in PTSD and the severity of each cluster (16). Difficulties in ER are not only associated with the severity of PTSD symptoms in a civilian sample (21); they also seem to play an important role in the chronification of PTSD in civilians (22). Other studies have shown positive effects for acceptance and reappraisal in a sample of veterans (17), and an effective treatment of PTSD can also reduce ER difficulties (23).

Furthermore, difficulties in ER might hinder the recovery from PTSD, as shown in investigations with civilians, although this result concerns the treatment phase (24). ER has not been investigated specifically in the context of PTSD in GAF service members. Thus far, only a pilot study has investigated the effect of emotional ambivalence on the occurrence of PTSD after deployment among GAF service members, but not ER. The results showed that higher emotional ambivalence connected to neuroticism leads to higher symptom severity (25). However, based on the literature, we hypothesized that it could be possible to generalize the relationship between ER and PTSD and that there could be a relationship between ER and PTSD in our sample as well.

Recent results showed that experiential avoidance mediates the association between PTSD symptoms and social support in veterans after deployment (26, 27). Experiential avoidance was examined according to the construct of psychological flexibility and measured by the Acceptance and Avoidance Questionnaire, which measures avoidance, acceptance, cognitive defusion, and mindfulness. These results suggest that there is potential importance in acceptance and action (AA) as a mediator of PTSD and related factors among deployed service members. Since some studies regard AA as part of the difficulties in ER, there is particular interest in its role as a mediator in this study (19).

Furthermore, Moral Injury (MI) seems to play an important role in the development of PTSD in service members (28–31). MI consists of shame and guilt resulting from a clash of prior beliefs and values with war experiences during deployment. Studies have reported on numerous situations that confront service members with ethically ambiguous situations created by modern warfare or deployment situations, such as shooting at enemies, being directly responsible for an enemy’s death, or seeing women and children wounded and being unable to help (32). Such situations may lead to MI (28).

For the subgroup of deployed GAF service members, this specific factor could possibly play a key role in the development of PTSD after foreign assignment with traumatic experiences. One therapeutic approach to MI consists of a value-based cognitive behavioral group therapy concept, which has shown promising results in a sample of GAF service members who suffer from PTSD (33). Studies investigating predictors of the development of PTSD in deployed service members of nations other than Germany have confirmed the importance of the MI construct and a therapeutic focus on it (28).

The MI concept has been examined in several studies in Germany following the work of international colleagues and their findings (28, 34). Previous findings among GAF service members after deployment show that MI constitutes a differential mediator between stressors (such as confrontation with hardship, suffering, and violence among the population in a war zone) and post-traumatic stress. Furthermore, according to a qualitative data analysis of structured interviews with veterans, veterans judge MI as an important war-related risk factor (35).

Among deployed GAF service members, MI has been shown to be a moderator between deployment-related stressors and PTSD, depression, and alcohol abuse (36). However, in a more recent study, the MI Event Scale (MIES) showed no significant difference between GAF service members with PTSD and those without it (36). According to that study, there was a mediating effect of MI on the relationship between certain factors and PTSD, but there was no significant difference between service members with and without PTSD in the specific population of GAF service members. Thus, the mediating effect of MI and ER on PTSD is a present interest (36).

PTSD is mostly associated with pathological aspects in civilians and service members, including chronic stress (37–39) or uncontrollable and recurring thoughts (40–42). In contrast, PTSD can be associated with positive psychological factors in civilians and military personnel, such as satisfaction with life (43–45) or post-traumatic growth as a coping strategy that helps people regain control by defining positive aspects of the traumatic experience (46). Recent studies have identified protective factors that are thematically independent from the military context and were found to be protective factors for the development of PTSD in civilians and deployed GAF service members. One example is psychological flexibility, which is the ability to remain focused on the present moment, even during a traumatic event. Psychological flexibility was shown to be a protective factor for the development of PTSD in both civilians and deployed GAF service members (47–49). Higher focus on hedonism and power (14) or hope and religiosity (50) have been identified as other protective factors for the development of PTSD.

There is strong evidence for the effects of social support during and after a traumatic event (51–53) and social acknowledgment (SA) as a victim or survivor (46, 54–57), which have both been repeatedly illustrated as potential resilience factors in civilians and veterans. On the other hand, a lack of social acknowledgment as a victim or survivor has repeatedly been shown to be a risk factor in terms of higher PTSD rates among veterans (54, 58, 59). Additionally, findings in military and civilian samples have shown that negative social reactions have a higher influence on PTSD than positive social reactions (54).

Based on the various findings from previous studies, the aim of the present study was to test the following hypotheses. Firstly, it was hypothesized that there is a direct relationship between ER and PTSD in our sample of deployed GAF service members. The second hypothesis was that the relationship between ER and PTSD is fully or partly mediated by one or more of the following three factors: MI, SA, and AA.

## Materials and Methods

### Participants

The participants (*N* = 72) were German men who spoke German as their native language. The mean age of the participants was 38.24 years (*SD* = 8.75 years; range: 19–70 years). **Table 1** provides demographic variables regarding their marital status, graduation, completion of training, employment status, and military branch, while **Table 2** presents data about the diagnosed mental disorders among the sample.

**Table 1:** Demographic characteristics of participants.

	Frequencies		Statistics
	PTSD (*n* = 25)	Non-PTSD *n* = 47	
Treatment-seeking	- yes: *n* = 25 - no: *n* = 0	- yes: *n* = 14 - no: *n* = 33	χ^2^(1, 72) = 32.41; *p* <.001
Marital status	- single without relationship: *n* = 3 - single with relationship: *n* = 3 - married: *n* = 12 - divorced: *n* = 5 - n. a.: *n* = 2	- single without relationship: *n* = 1 - single with relationship: *n* = 13 - married: *n* = 26 - divorced: *n* = 6 - n. a.: *n* = 1	χ^2^(1, 69) = 5.44; *p* = .143
Graduation	- Primary school: *n* = 5 - Intermediate school leaving certificate: *n* = 14 - Vocational baccalaureate diploma: *n* = 3 - A-levels: *n* = 2 - n. a.: *n* = 1	- Primary school: *n* = 3 - Intermediate school leaving certificate: *n* = 22 - Vocational baccalaureate diploma: *n* = 10 - A-levels: *n* = 12	χ^2^(1, 71) = 8.84, *p* = .183
Completion of training	- No vocational qualification: *n* = 3 - In vocational training: *n* = 0 - Completed vocational training: *n* = 13 - Technical college degree: *n* = 4 - Bachelor degree: *n* = 0 - Master degree in technical college: *n* = 1 - Master degree from an university: *n* = 2 - n. a.: *n* = 2	- No vocational qualification: *n* = 3 - In vocational training: *n* = 2 - Completed vocational training: *n* = 21 - Technical college degree: *n* = 8 - Bachelor degree: *n* = 2 - Master degree in technical college: *n* = 3 - Master degree from am university: *n* = 6 - n. a.: *n* = 2	χ^2^(1, 70) = 4.52, *p* = .719
Employment status	- Voluntary military service: *n* = 1 - Soldier for a fixed term: *n* = 13 - Professional soldier: *n* = 5 - Service status in special form: *n* = 1 - n. a.: *n* = 5	- Voluntary military service: *n* = 1 - Soldier for a fixed term: *n* = 18 - Professional soldier: *n* = 26 - Service status in special form: *n* = 2	χ^2^(1, 67) = 5.36, *p* = .148
Military branch	- Army: *n* = 11 - German Air Force: *n* = 4 - Navy: *n* = 0 - Medical Service: *n* = 3 - Joint support service: *n* = 5 - n. a.: *n* = 2	- Army: *n* = 15 - German Air Force: *n* = 15 - Navy: *n* = 2 - Medical Service: *n* = 4 - Joint support service: *n* = 11	χ^2^(1, 70) = 3.57, *p* = .468
Service grade	- Ratings: *n* = 6 - Non-commissioned officer: *n* = 15 - Officer: *n* = 2 - n. a.: *n* = 2	- Ratings: *n* = 8 - Non-commissioned officer: *n* = 27 - Officer: *n* = 12	χ^2^(1, 70) = 2.98, *p* = .226

n. a., not available.

**Table 2:** Clinical data of the PTSD (*n* = 25) and Non-PTSD group (*n* = 47).

	PTSD	Non-PTSD	Statistics
Current major depressive disorder	*n* = 12 (48.0%)	*n* = 2 (4.3%)	χ^2^(1, 72) = 19.937, *p* <.001
Current panic disorder	*n* = 9 (36.0%)	*n* = 0 (0.0%)	χ^2^(1, 72) = 19.337, *p* <.001
Current agoraphobia	*n* = 17 (68.0%)	*n* = 4 (8.5%)	χ^2^(1, 72) = 27.955, *p* <.001
Current social anxiety disorder	*n* = 7 (28.0%)	*n* = 0 (0.0%)	χ^2^(1, 72) = 14.577, *p* <.001
Current generalized anxiety disorder	*n* = 5 (20.0%)	*n* = 1 (2.1%)	χ^2^(1, 72) = 6.824, *p* = .009
Current suicidality	*n* = 4 (16.0%)	*n* = 1 (2.1%)	χ^2^(1, 72) = 4.860, *p* = .029
Lifetime suicide attempt	*n* = 5 (20.0%)	*n* = 1 (2.1%)	χ^2^(1, 72) = 6.824, *p* = .009
Current medical treatment	*n* = 11 (45.8%)	*n* = 6 (12.8%)	χ^2^(1,71) = 9.539, *p* = .002
Current psychiatric/psychotherapeutic treatment	*n* = 8 (33.3%)	*n* = 2 (4.3%)	χ^2^(1,71) = 11.101, *p* = .001
Current somatic disorder	*n* = 7 (29.2%)	*n* = 10 (21.3%)	χ^2^(1,71) = .543, *p* = .559
Regular use of medication	*n* = 13 (54.2%)	*n* = 12 (25.5%)	χ^2^(1,71) = .5.710, *p* = .018

### Study Design and Sampling Procedure

Data were collected between July 2016 and July 2018. Data from evaluation questionnaires administered upon entry into the study were subjected to a cross-sectional analysis. The inclusion criteria were status as an active or former service member of the GAF, male sex, and meeting criterion A according to DSM-5 for PTSD after having been deployed. The exclusion criteria were acute psychotic symptoms, an acute manic episode, current substance abuse or dependence, an acute high risk of suicide, neurological disorder, acute somatic disease, unstable psychotropic medication, or concurrent psychotherapeutic treatment.

In a quasi-experimental design, participants were separated into two groups according to the PTSD diagnosis based on the Clinician-Administered PTSD Scale for DSM-5 (CAPS-5). Overall, *N* = 89 service members were screened. Participants without deployment and those with incomplete CAPS-5 scores were excluded. Accordingly, *n* = 39 treatment-seeking GAF service members and *n* = 33 GAF service members from the control group of the original RCT (60) were pooled (*n* = 72) and subsequently allocated to either the PTSD or the non-PTSD group according to their CAPS-5 PTSD diagnosis. Ultimately, a total of *n* = 25 participants fulfilled the PTSD criteria (PTSD group), while *n* = 47 participants experienced a traumatic event but did not fulfill the PTSD criteria (non-PTSD group).

Participating service members were deployed one or more times. More than half of the participants served in Afghanistan (58.9%), whereas 20.6% of the participants served in Kosovo, and 7.4% served in Mali. There were no significant differences between the missions (χ²(**40**) = 38.358, *p* = 0.544). The time since deployment varied between six weeks and 26 years (*M* = 7.0, *SD* = 5.4) and did not differ significantly between groups [*U*(*N_PTSD_* = 20, *N_non-PTSD_ =* 45) = 320.0, z = −1.848, *p* = 0.065]. A detailed description of the procedure of the initial study is available elsewhere (60).The traumatic events experienced by both groups were measured with the Life Events Checklist for DSM-5 (61). As shown in **Table 3**, the frequencies of traumatic events did not differ significantly between groups.

**Table 3:** Frequencies of traumatic events according to the Life Events Checklist for DSM-5 for GAF service members with and without PTSD.

Traumatic event	PTSD	Non-PTSD	Statistics
Natural disaster	- directly experienced: *n* = 4 - witnessed: *n* = 5 - learned about it: *n* = 1 - part of job: *n* = 2 - not sure: *n* = 0 - doesn’t apply: *n* = 12 - n. a.: *n* = 1	- directly experienced: *n* = 8 - witnessed: *n* = 7 - learned about it: *n* = 7 - part of job: *n* = 8 - not sure: *n* = 1 - doesn’t apply: *n* = 16 - n. a.: *n* = 0	χ^2^(5, 71) = 4.343, *p* = .501
Fire or explosion	- directly experienced: *n* = 9 - witnessed: *n* = 6 - learned about it: *n* = 3 - part of job: *n* = 3 - not sure: *n* = 0 - doesn’t apply: *n* = 3 - n. a.: *n* = 1	- directly experienced: *n* = 10 - witnessed: *n* = 12 - learned about it: *n* = 5 - part of job: *n* = 9 - not sure: *n* = 0 - doesn’t apply: *n* = 10 - n. a.: *n* = 1	χ^2^(5, 70) = 2.671, *p* = .614
Transportation accident	- directly experienced: *n* = 10 - witnessed: *n* = 6 - learned about it: *n* = 1 - part of job: *n* = 2 - not sure: *n* = 0 - doesn’t apply: *n* = 5 - n. a.: *n* = 1	- directly experienced: *n* = 28 - witnessed: *n* = 10 - learned about it: *n* = 4 - part of job: *n* = 2 - not sure: *n* = 0 - doesn’t apply: *n* = 3 - n. a.: *n* = 0	χ^2^(5, 71) = 4.889, *p* = .299
Serious accident at work, home, or during recreational activity	- directly experienced: *n* = 4 - witnessed: *n* = 4 - learned about it: *n* = 3 - part of job: *n* = 1 - not sure: *n* = 2 - doesn’t apply: *n* = 10 - n. a.: *n* = 1	- directly experienced: *n* = 10 - witnessed: *n* = 10 - learned about it: *n* = 11 - part of job: *n* = 4 - not sure: *n* = 0 - doesn’t apply: *n* = 12 - n. a.: *n* = 0	χ^2^(5, 71) = 6.978, *p* = .222
Exposure to toxic substance	- directly experienced: *n* = 2 - witnessed: *n* = 0 - learned about it: *n* = 0 - part of job: *n* = 3 - not sure: *n* = 2 - doesn’t apply: *n* = 17 - n. a.: *n* = 1	- directly experienced: *n* = 7 - witnessed: *n* = 2 - learned about it: *n* = 3 - part of job: *n* = 7 - not sure: *n* = 2 - doesn’t apply: *n* = 26 - n. a.: *n* = 0	χ^2^(5, 71) = 4.258, *p* = .513
Physical assault	- directly experienced: *n* = 8 - witnessed: *n* = 4 - learned about it: *n* = 2 - part of job: *n* = 0 - not sure: *n* = 1 - doesn’t apply: *n* = 9 - n. a.: *n* = 1	- directly experienced: *n* = 18 - witnessed: *n* = 7 - learned about it: *n* = 7 - part of job: *n* = 0 - not sure: *n* = 1 - doesn’t apply: *n* = 14 - n. a.: *n* = 0	χ^2^(5, 71) = 1.205, *p* = .877
Assault with a weapon	- directly experienced: *n* = 14 - witnessed: *n* = 2 - learned about it: *n* = 1 - part of job: *n* = 4 - not sure: *n* = 0 - doesn’t apply: *n* = 3 - n. a.: *n* = 1	- directly experienced: *n* = 15 - witnessed: *n* = 3 - learned about it: *n* = 6 - part of job: *n* = 3 - not sure: *n* = 1 - doesn’t apply: *n* = 19 - n. a.: *n* = 0	χ^2^(5, 71) = 10.205, *p* = .070
Sexual assault	- directly experienced: *n* = 0 - witnessed: *n* = 0 - learned about it: *n* = 1 - part of job: *n* = 1 - not sure: *n* = 0 - doesn’t apply: *n* = 22 - n. a.: *n* = 1	- directly experienced: *n* = 1 - witnessed: *n* = 1 - learned about it: *n* = 7 - part of job: *n* = 0 - not sure: *n* = 0 - doesn’t apply: *n* = 38 - n. a.: *n* = 0	χ^2^(5, 71) = 4.822, *p* = .306
Other unwanted or uncomfortable sexual experience	- directly experienced: *n* = 0 - witnessed: *n* = 0 - learned about it: *n* = 0 - part of job: *n* = 0 - not sure: *n* = 3 - doesn’t apply: *n* = 21 - n. a.: *n* = 1	- directly experienced: *n* = 2 - witnessed: *n* = 0 - learned about it: *n* = 5 - part of job: *n* = 0 - not sure: *n* = 1 - doesn’t apply: *n* = 39 - n. a.: *n* = 0	χ^2^(5, 71) = 6.647, *p* = .084
Combat or exposure to a war-zone	- directly experienced: *n* = 16 - witnessed: *n* = 1 - learned about it: *n* = 0 - part of job: *n* = 4 - not sure: *n* = 1 - doesn’t apply: *n* = 2 - n. a.: *n* = 1	- directly experienced: *n* = 29 - witnessed: *n* = 1 - learned about it: *n* = 3 - part of job: *n* = 8 - not sure: *n* = 1 - doesn’t apply: *n* = 5 - n. a.: *n* = 0	χ^2^(5, 71) = 2.149, *p* = .828
Captivity	- directly experienced: *n* = 0 - witnessed: *n* = 0 - learned about it: *n* = 1 - part of job: *n* = 1 - not sure: *n* = 0 - doesn’t apply: *n* = 22 - n. a.: *n* = 1	- directly experienced: *n* = 0 - witnessed: *n* = 1 - learned about it: *n* = 4 - part of job: *n* = 0 - not sure: *n* = 1 - doesn’t apply: *n* = 41 - n. a.: *n* = 0	χ^2^(5, 71) = 3.440, *p* = .487
Life-threatening illness or injury	- directly experienced: *n* = 2 - witnessed: *n* = 5 - learned about it: *n* = 2 - part of job: *n* = 1 - not sure: *n* = 2 - doesn’t apply: *n* = 12 - n. a.: *n* = 1	- directly experienced: *n* = 2 - witnessed: *n* = 17 - learned about it: *n* = 9 - part of job: *n* = 0 - not sure: *n* = 0 - doesn’t apply: *n* = 19 - n. a.: *n* = 0	χ^2^(5, 71) = 9.083, *p* = .106
Severe human suffering	- directly experienced: *n* = 3 - witnessed: *n* = 10 - learned about it: 0 - part of job: *n* = 4 - not sure: *n* = 2 - doesn’t apply: *n* = 4 - n. a.: *n* = 2	- directly experienced: *n* = 6 - witnessed: *n* = 19 - learned about it: *n* = 3 - part of job: *n* = 10 - not sure: *n* = 2 - doesn’t apply: *n* = 7 - n. a.: *n* = 0	χ^2^(5, 70) = 2.214, *p* = .819
Sudden violent death	- directly experienced: *n* = 3 - witnessed: *n* = 9 - learned about it: *n* = 2 - part of job: *n* = 2 - not sure: *n* = 1 - doesn’t apply: *n* = 7 - n. a.: *n* = 1	- directly experienced: *n* = 4 - witnessed: *n* = 5 - learned about it: *n* = 16 - part of job: *n* = 2 - not sure: *n* = 2 - doesn’t apply: *n* = 18 - n. a.: *n* = 0	χ^2^(5, 71) = 11.058, *p* = .050
Sudden accidental death	- directly experienced: *n* = 1 - witnessed: *n* = 3 - learned about it: *n* = 3 - part of job: *n* = 1 - not sure: *n* = 1 - doesn’t apply: *n* = 15 - n. a.: *n* = 1	- directly experienced: *n* = 1 - witnessed: *n* = 7 - learned about it: *n* = 15 - part of job: *n* = 4 - not sure: *n* = 2 - doesn’t apply: *n* = 18 - n. a.: *n* = 0	χ^2^(5, 71) = 5.089, *p* = .405
Serious injury, harm, or death caused to someone else	- directly experienced: *n* = 4 - witnessed: *n* = 0 - learned about it: *n* = 1 - part of job: *n* = 2 - not sure: *n* = 1 - doesn’t apply: *n* = 16 - n. a.: *n* = 1	- directly experienced: *n* = 5 - witnessed: *n* = 0 - learned about it: *n* = 0 - part of job: *n* = 0 - not sure: *n* = 1 - doesn’t apply: *n* = 41 - n. a.: *n* = 0	χ^2^(5, 71) = 7.402, *p* = .116
Any other very stressful event or experience	- directly experienced: *n* = 14 - witnessed: *n* = 0 - learned about it: *n* = 0 - part of job: *n* = 3 - not sure: *n* = 2 - doesn’t apply: *n* = 4 - n. a.: *n* = 2	- directly experienced: *n* = 14 - witnessed: *n* = 2 - learned about it: *n* = 1 - part of job: *n* = 2 - not sure: *n* = 5 - doesn’t apply: *n* = 21 - n. a.: *n* = 2	χ^2^(5, 68) = 9.972, *p* = .076

n. a., not available.

The PTSD group showed a mean CAPS-5 sum score of 42.52 (*SD* = 11.62; range: 21–62), whereas that of the non-PTSD group was significantly lower at 7.79 (*SD* = 10.94; range: 0–42) [*U*(*N_PTSD_* = 25, *N_non-PTSD_ =* 47) = 31.5, z = −6.650, *p* < 0.001]. The groups did not significantly differ in age [*U*(*N_PTSD_* = 24, *N_non-PTSD_ =* 47) = 496.5, z = −0.821, *p* = 0.411], number of people living in their households [*U*(*N_PTSD_* = 23, *N_non-PTSD_ =* 45) = 478.0, z = −0.528, *p* = 0. 598], number of children [*U*(*N_PTSD_* = 24, *N_non-PTSD_ =* 47) = 513.5, z = −0.639, *p* = 0.523], number of international assignments [*U*(*N_PTSD_* = 24, *N_non-PTSD_ =* 47) = 505.0, z = −0.735, *p* = 0.462], or length of international assignments [*U*(*N_PTSD_* = 20, *N*_*non-PTSD*_ = 45) = 432.5, z = −0.249, *p* = 0.803]. However, net income was significantly lower in the PTSD group than the non-PTSD group (*p* = 0.003). As shown in **Table 1**, the groups did not differ significantly regarding other demographic variables. However, as illustrated in **Table 2**, the PTSD group showed significantly higher rates of mental disorders than the non-PTSD group.

### Measures

#### Clinician-Administered PTSD Scale for DSM-5 (CAPS-5)

The PTSD diagnosis and symptom severity were assessed with the German translation of the CAPS-5 (62). The CAPS-5 is a structured clinical diagnostic interview for the assessment of PTSD based on the criteria of DSM-5 (2). The original version of CAPS-5 shows good psychometric properties with an internal consistency (Cronbach’s α) of α = 0.88 and good convergent validity with the CAPS-4 severity score with *r* = 0.83. The CAPS-5 also shows high correlations with self-rated scales that measure PTSD symptoms according to DSM-5 (*r* = 0.66) (63). The German version is currently being validated (64).

#### Difficulties in Emotion Regulation Scale (DERS)

The DERS was used to evaluate the severity of deficits in ER (65). The scale has 36 items with a five-point Likert scale ranging from 1 = “almost never” to 5 = “almost always” (the total score ranges from 36 to 180, with higher scores indicating more difficulties in ER). This self-rated questionnaire assesses six factors of ER strategies: “nonacceptance,” “goals,” “impulse,” “awareness,” “strategies,” and “clarity.” The DERS shows high internal consistencies for the subscales with α = 0.82–0.92 and an overall internal consistency of α = 0.95 (65, 66).

#### Acceptance and Action Questionnaire–II (AAQ-II)

The AAQ-II (67) measures the construct of psychological flexibility. Psychological flexibility is defined as a superordinate construct consisting of avoidance, acceptance, cognitive defusion, and mindfulness. Items are rated on a seven-point scale from 0 = “never true” to 7 = “always true.” A higher score reflects lower psychological flexibility (67). The original version has good internal consistency with α = 0.84 and test-retest reliability with *r_tt_* between 0.81 (3 months) and 0.79 (12 months) (67). For the German version of the AAQ-II, excellent internal consistency of α = 0.97 was found in a student sample, and good internal consistency was found in a clinical sample with α = 0.84 (68).

#### Moral Injury Event Scale (MIES)

The MIES (36, 69) is a self-rated questionnaire that measures the burden of events that violate deeply rooted moral beliefs and values. Items are assessed on a six-point Likert scale (0 = “strongly agree” to 5 = “strongly disagree”). It has nine items in total, which are split between two factors: “perceived transgressions by self or others” (six items) and “perceived betrayals by others, inside or outside the military” (three items) (69). The internal consistency of the German version was α = 0.82 for the first subscale and α = 0.78 for the second subscale (36).

#### The Post-Traumatic Cognitions Inventory (PTCI)

The PTCI (70) is used to identify dysfunctional cognitions that play a key role in the development and persistence of PTSD. This self-rated questionnaire consists of 33 items that are answered on a seven-point Likert scale from 1= “totally disagree” to 7 = “totally agree” (range: 33 to 231). The three subscales are “negative cognitions about the world,” “negative cognitions about oneself,” and “self-blame,” which show good internal consistency values of α = 0.86–0.97 and an overall consistency of α = 0.97 (70).

#### White Bear Suppression Inventory (WBSI)

The WBSI focuses on the experience of uncontrollable and recurring thoughts, as well as the desire and attempt to suppress these thoughts through avoidance and distraction. The original version has shown good internal consistency with Cronbach’s α = 0.87–0.89 in five different samples (71). It also has high test-retest reliability (*r_tt_* = 0.86; interval between 5 days and 5 weeks) (71). The German version has a good internal consistency of α = 0.88 and a satisfactory test-retest correlation of *r_tt_* = 0.78 after 3–6 weeks (72).

#### Satisfaction With Life Scale (SWLS)

The SWLS consists of five items and measures global cognitive judgments of one’s life satisfaction as a whole. Items are rated on a seven-point Likert scale from 1 = “strongly agree” to 7 = “strongly disagree” (range 5 to 35). A higher score reflects a lower satisfaction with life (73). The internal consistency varies between studies in the range of α = 0.86–0.89 (73, 74).

#### Post-Traumatic Growth Inventory (PTGI)

The PTGI assesses post-traumatic growth reported by people who have experienced traumatic events (75). Post-traumatic growth is defined as how successful individuals cope with the aftermath of trauma and reconstruct or strengthen their perceptions of themselves, others, and the meaning of events. The PTGI uses 21 items with five subscales: “relating to others,” “new possibilities,” “personal strength,” “spiritual change,” and “appreciation of life.” The answers are rated from 0 = “I did not experience this change as a result of my crisis” to 5 = “I experienced this change to a very great degree as a result of my crisis” (range: 0–105). A higher total score means that more post-traumatic growth has occurred. The internal consistency of the total score of the PTGI is α = 0.94 (76).

#### Crisis Support Scale (CSS)

Social support was determined by using the CSS (77). This self-rated questionnaire has 14 items, which are each rated on a seven-point Likert scale ranging from 1 = “never” to 7 = “always.” The first six items were asked twice to measure crisis support directly following a traumatic event (T1) and at the present time (T2). The seventh item measures the satisfaction with overall crisis support at T1 and T2. The total score varies between 6 and 42 for each subscale, and a higher score indicates a higher level of support. The internal consistencies of the subscales are α = 0.6–0.75 at T1, α = 0.67–0.69 at T2, and α = 0.82 for the entire scale (77, 78).

#### Social Acknowledgment as a Victim or Survivor Questionnaire (SAQ)

The SAQ is a self-rated questionnaire that assesses social acknowledgment as a victim or survivor. The SAQ asks for the degree to which people feel validated and supported by their social environment following a traumatic event. It comprises 16 items in three subscales that are rated on a six-point Likert scale from 0 = “denial” to 5 = “agreement.” The SAQ measures three factors of social acknowledgment: “recognition as a victim,” “general disapproval,” and “family disapproval.” The internal consistency is α = 65 for the recognition subscale, α = 0.79 for the general disapproval subscale, α = 0.80 for the family disapproval subscale, and α = 0.75 for the SAQ sum score (55).

### Statistical Analysis

The data were analyzed using SPSS version 25.0 for macOS (79). Descriptive data are presented as frequencies (%), mean scores, and standard deviations. The Shapiro-Wilk test results showed that the data of all variables were not normally distributed except for the SAQ (*p* = 0.246), so methods for the analysis of non-parametric data were used. In the first step, associations of the severity of PTSD symptoms and clusters of PTSD symptoms (intrusions, avoidance, negative alterations in cognitions and mood, and hyperarousal measured with CAPS-5) with clinical measures were analyzed with the Spearman score correlation coefficient (*r_s_*) for the whole sample (*N* = 72).

In the next step, differences between groups were analyzed using X^2^ tests for nominal data and Mann-Whitney U-tests for non-parametric data. Eta-squared (η^2^) was calculated as an effect-size estimator of the differences between mean scores in the Mann-Whitney U-tests. η^2^ ≥ 0.01 indicates a small effect, η^2^ ≥ 0.06 indicates a medium effect, and η^2^ ≥ 0.14 indicates a large effect. Due to the exploratory nature of the data analysis, no corrections for multiple comparisons were conducted regarding the between-group analyses.

This study pooled treatment-seeking GAF service members and GAF service members in the control group of the original RCT who were not seeking treatment. Subsequently, all GAF service members were allocated to a PTSD and non-PTSD group, and *n* = 14 GAF service members who were seeking treatment were allocated to the non-PTSD group because they did not fulfill the PTSD criteria according to the CAPS-5 (see **Table 1**). Thus, a sensitivity analysis was conducted without these 14 GAF service members (PTSD group: *n* = 25; 100% treatment seeker; non-PTSD group: *n* = 33; 0% treatment seekers).

Finally, to test our hypotheses, a mediation analysis was chosen with an empirical approach, and variables were selected according to the literature (80). The mediation analyses were performed using the PROCESS macro by Hayes, which uses ordinary least squares regression and yields unstandardized path coefficients for total, direct, and indirect effects (81).

Bootstrapping with 5,000 samples together with heteroscedasticity consistent standard errors were used to compute the confidence intervals and inferential statistics (82). Effects were deemed significant when the confidence interval did not include zero (81). The relationship of all variables involved in the mediation analysis was linear according to the visual inspection of scatterplots after LOESS smoothing, and the residuals were normally distributed (81).

## Results

The non-parametric correlation analyses showed that the severity of PTSD symptoms (measured with the CAPS-5 sum score) and all clusters of PTSD symptoms (intrusions, avoidance, negative alterations in cognitions and mood, and hyperarousal) were significantly associated with most of the measured constructs. Only PTGI showed no significant associations with the severity of PTSD symptoms and clusters of PTSD. The results of the correlation analyses showed associations between constructs in expectable directions. The severity of PTSD symptoms and the symptoms themselves showed significant positive associations with constructs measuring psychopathology. However, the correlation analyses with constructs measuring resilience and positive psychological constructs showed significant negative associations with the symptoms and their severity (see **Table 4**).

**Table 4:** Spearman rank-correlations between CAPS-5 sum score (B+C+D+E), CAPS-5 subscale scores, and criteria measures.

	DERS	AAQ-II	SAQ	MIES	SWLS	CSS	PTCI	PTGI	WBSI
CAPS sum score	.863^***^ (*n* = 68)	.877^***^ (*n* = 70)	–.798^***^ (*n* = 66)	.455^***^ (*n* = 66)	.778^***^ (*n* = 70)	–.794^***^ (*n* = 69)	.867^***^ (*n* = 68)	.022 (*n* = 67)	.834^***^ (*n* = 70)
CAPS-B	.799^***^ (*n* = 68)	.822^***^ (*n* = 70)	–.745^***^ (*n* = 66)	.400^** ^(*n* = 66)	.719^***^ (*n* = 70)	–.736^***^ (*n* = 69)	.791^***^ (*n* = 68)	.033 (*n* = 67)	.756^***^ (*n* = 70)
CAPS-C	.822^***^ (*n* = 68)	.803^***^ (*n* = 70)	–.752^***^ (*n* = 66)	.389^**^ (*n* = 66)	.756^***^ (*n* = 70)	–.733^***^ (*n* = 69)	.801^***^ (*n* = 68)	.005 (*n* = 67)	.834^***^ (*n* = 70)
CAPS-D	.836^***^ (*n* = 68)	.842^***^ (*n* = 70)	–.755^***^ (*n* = 66)	.474^**^ (*n* = 66)	.793^***^ (*n* = 70)	–.759^***^ (*n* = 69)	.868^***^ (*n* = 68)	–.056 (*n* = 67)	.794^***^ (*n* = 70)
CAPS-E	.834^***^ (*n* = 68)	.853^***^ (*n* = 70)	–.753^***^ (*n* = 66)	.422^**^ (*n* = 66)	.749^***^ (*n* = 70)	–.779^***^ (*n* = 69)	.812^***^ (*n* = 68)	–.003 (*n* = 67)	.799^***^ (*n* = 70)

CAPS, Clinician-Administered PTSD scale; CAPS-B, intrusions subscale of the CAPS; CAPS-C, avoidance subscale of the CAPS; CAPS-D, negative alterations in cognitions and mood subscale of the CAPS; CAPS-E, hyperarousal subscale of the CAPS; CSS, crisis support scale; DERS, difficulties in emotion regulation scale; AAQ-II, acceptance and action questionnaire–II; PTCI, posttraumatic cognitions questionnaire; PTGI, posttraumatic growth inventory; SAQ, social acknowledgment as a victim or survivor questionnaire; MIES, moral injury event scale; SWLS, satisfaction with life scale; WBSI, white bear suppression inventory; **p < 0.01; ***p < 0.001.

Next, differences in mean scores of the measures between groups were analyzed. As illustrated in **Table 5**, the PTSD group showed significantly higher mean scores on questionnaires measuring factors that have been associated with the psychopathology of PTSD. However, this group showed significantly lower mean scores in social support (CSS) and social acknowledgment as a victim or survivor questionnaire (SAQ) than the non-PTSD group. In accordance with the correlation analysis, the groups did not differ significantly in the mean scores of the PTGI. These analyses were repeated after the exclusion of *n* = 14 treatment-seeking GAF service members, and the results were in a comparable range with slightly larger effect sizes (see **Table 6**).

**Table 5:** Results of Mann-Whitney-U-Tests regarding differences of mean ranks of measured questionnaires between service members with PTSD (*n* = 25) and service members without PTSD (*n* = 47; including treatment seekers).

	*Mean Rank*		*Statistics*
	*PTSD*	*Non-PTSD*	
CAPS sum score	*Mdn* = 58.74	*Mdn* = 24.67	*U*(*N_PTSD_* = 25, *N_Non-PTSD_ =* 47) = 31.5, z = –6.65, *p* <.001; η^2^ = .601
DERS	*Mdn* = 53.73	*Mdn* = 24.01	*U*(*N_PTSD_* = 24, *N_Non-PTSD_ =* 44) = 66.5, z = –5.92, *p* <.001; η^2^ = .516
AAQ-II	*Mdn* = 54.63	*Mdn* = 25.52	*U*(*N_PTSD_* = 24, *N_Non-PTSD_ =* 46) = 93.0, z = –5.69, *p* <.001; η^2^ = .461
SAQ	*Mdn* = 16.70	*Mdn* = 42.49	*U*(*N_PTSD_* = 23, *N_Non-PTSD_ =* 43) = 108.0, z = –5.21, *p* <.001; η^2^ = .410
MIES	*Mdn* = 40.61	*Mdn* = 29.94	*U*(*N_PTSD_* = 22, *N_Non-PTSD_ =* 44) = 327.5, z = –2.13, *p* = .033; η^2^ = .069
SWLS	*Mdn* = 53.63	*Mdn* = 26.04	*U*(*N_PTSD_* = 24, *N_Non-PTSD_ =* 46) = 117.0, z = –5.39, *p* <.001; η^2^ = .414
CSS	*Mdn* = 28.04	*Mdn* = 38.48	*U*(*N_PTSD_* = 23, *N_Non-PTSD_ =* 46) = 369.0, z = –2.08, *p* = .037; η^2^ = .060
PTCI	*Mdn* = 52.04	*Mdn* = 24.93	*U*(*N_PTSD_* = 24, *N_Non-PTSD_ =* 44) = 107.0, z = –5.40, *p* <.001; η^2^ = .429
PTGI	*Mdn* = 32.28	*Mdn* = 34.90	*U*(*N_PTSD_* = 23, *N_Non-PTSD_ =* 44) = 466.5, z = –.52, *p* = .602; η^2^ = .004
WBSI	*Mdn* = 54.48	*Mdn* = 25.60	*U*(*N_PTSD_* = 24, *N_Non-PTSD_ =* 46) = 96.5, z = –5.64, *p* <.001; η^2^ = .454

Mdn, median; PTSD, posttraumatic stress disorder; CAPS, Clinician-Administered PTSD scale; CSS, crisis support scale; DERS, difficulties in emotion regulation scale; AAQ-II, acceptance and action questionnaire–II; PTCI, posttraumatic cognitions questionnaire; PTGI, posttraumatic growth inventory; SAQ, social acknowledgment as a victim or survivor questionnaire; MIES, moral injury event scale; SWLS, satisfaction with life scale; WBSI, white bear suppression inventory.

**Table 6:** Results of Mann-Whitney-U-Tests regarding differences of mean ranks of measured questionnaires between service members with PTSD (*n* = 25) and service members without PTSD (*n* = 33; excluding treatment seekers).

	*Mean Rank*		*Statistics*
	*PTSD*	*Non-PTSD*	
CAPS sum score	*Mdn* = 46.00	*Mdn* = 17.00	*U*(*N_PTSD_* = 25, *N_Non-PTSD_ =* 33) = 0.0, z = –6.62, *p* <.001; η^2^ = .723
DERS	*Mdn* = 42.42	*Mdn* = 15.57	*U*(*N_PTSD_* = 24, *N_Non-PTSD_ =* 30) = 2.0, z = –6.23, *p* <.001; η^2^ = .719
AAQ-II	*Mdn* = 44.77	*Mdn* = 17.53	*U*(*N_PTSD_* = 24, *N_Non-PTSD_ =* 33) = 17.5, z = –6.14, *p* <.001; η^2^ = .657
SAQ	*Mdn* = 13.22	*Mdn* = 37.57	*U*(*N_PTSD_* = 23, *N_Non-PTSD_ =* 30) = 28.0, z = –5.70, *p* <.001; η^2^ = .611
MIES	*Mdn* = 34.43	*Mdn* = 20.68	*U*(*N_PTSD_* = 22, *N_Non-PTSD_ =* 30) = 155.5, z = –3.24, *p* = .001; η^2^ = .201
SWLS	*Mdn* = 43.54	*Mdn* = 17.22	*U*(*N_PTSD_* = 24, *N_Non-PTSD_ =* 32) = 23.0, z = –5.99, *p* <.001; η^2^ = .638
CSS	*Mdn* = 20.48	*Mdn* = 33.41	*U*(*N_PTSD_* = 23, *N_Non-PTSD_ =* 32) = 195.0, z = –3.03, *p* = .002; η^2^ = .158
PTCI	*Mdn* = 41.38	*Mdn* = 16.40	*U*(*N_PTSD_* = 24, *N_Non-PTSD_ =* 30) = 27.0, z = –5.80, *p* <.001; η^2^ = .622
PTGI	*Mdn* = 26.52	*Mdn* = 28.23	*U*(*N_PTSD_* = 23, *N_Non-PTSD_ =* 31) = 334.0, z = –.39, *p* = .694; η^2^ = .003
WBSI	*Mdn* = 44.71	*Mdn* = 17.58	*U*(*N_PTSD_* = 24, *N_Non-PTSD_ =* 33) = 19.0, z = –6.10, *p* <.001; η^2^ = .651

Mdn, median; PTSD, posttraumatic stress disorder; CAPS, Clinician-Administered PTSD scale; CSS, crisis support scale; DERS, difficulties in emotion regulation scale; AAQ-II, acceptance and action questionnaire–II; PTCI, posttraumatic cognitions questionnaire; PTGI, posttraumatic growth inventory; SAQ, social acknowledgment as a victim or survivor questionnaire; MIES, moral injury event scale; SWLS, satisfaction with life scale; WBSI, white bear suppression inventory.

Finally, a simple analysis for parallel mediation was performed to determine whether there is a relationship between ER and PTSD (measured with CAPS subtotal score) and whether the direct path is mediated by MI (measured with the MIES), AA (measured with AAQ-II), and SA (measured by the SAQ). A relationship between ER and PTSD was observed (*B* = 21.764, *p* < 0.001). After entering the three mediators into the model, there was a significant relationship between ER and the mediator MI (*B* = 3.833, *p* < 0.05), which in turn was not associated significantly with PTSD (*B* = 0.1844, *p* = 0.184).

**Figure 1:**
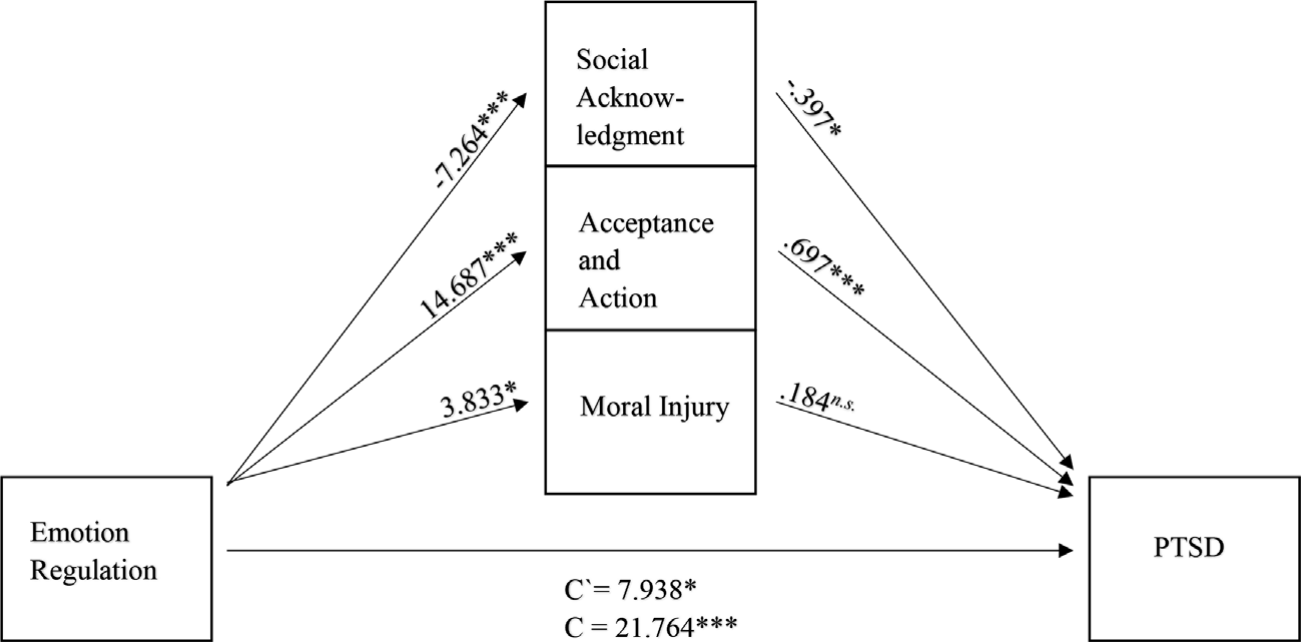
Mediation model (*N* = 72), with standardized beta weights and significant level for the relationship between ER and PTSD, mediated by SA, AA, and MI. 95%-*CI*, 95%-Confidence interval; Emotion Regulation measured by the DERS, Difficulties in Emotion Regulation Scale; Social Acknowledgment measured by the SAQ, Social Acknowledgment as a Victim or Survivor Questionnaire; Acceptance and Action measured by the AAQ-II, Acceptance and Action Questionnaire-II; Moral injury measured by the MIES, Moral Injury Event Scale; PTSD measured by the CAPS-5; *n.s.*, not significant; *p <.05; ***p < 0.001.

In contrast, there was a significant relationship between ER and the mediator AA, *B* = 14.687, *p* < 0.001, which in turn was significantly associated with PTSD (*B* = 0.697, *p* = 0.001). Additionally, there was a significant relationship between ER and the mediator SA (*B *= −7.264, *p *< 0.001), which in turn was significantly associated with PTSD (*B* = −0.397, *p* = 0.05). Finally, the results showed that the relationship between ER and PTSD is partially mediated by AA (indirect effect *ab* = 10.238, 95% CI [4.973, 16.300]) and by SA (*ab* = 2.880, 95% CI [−0.178, 5.306]), but not by MI (*ab* = 0.707, 95% CI [−0.551, 2.742], with an indirect effect total; *ab* = 13.825, 95% CI [7.592, 21.037]) (see **Figure 1**).

## Discussion

The aim of the present study was to determine the relationship between ER and the severity of PTSD symptoms in GAF service members, as well as possible mediating factors. Firstly, nonparametric correlation analyses revealed significant associations of the severity of PTSD symptoms as well as PTSD symptoms themselves with most of the measured constructs. Given that dissociation and post-traumatic cognitions are part of the PTSD diagnosis, significant positive associations were expected between the PTCI with PTSD symptoms and their severity. The experience of reoccurring uncontrollable thoughts and attempts to suppress the trauma-associated thoughts as part of the PTSD symptomatology indicated a significant positive association between the WBSI and PTSD symptoms and their severity. Furthermore, the positive associations between PTSD symptoms and their severity were expectable due to the fact that PTSD has been repeatedly associated with hyperactivation of the hypothalamic-pituitary-adrenal axis (83, 84).

In line with previous research on veterans, MI (31, 35, 85) and difficulties with ER (19, 86, 87) also showed significant positive associations with PTSD symptoms and their severity in GAF service members. Furthermore, there were significant associations of PTSD symptoms and their severity in this sample with resilience factors that have repeatedly been associated with lower PTSD symptoms in veterans, such as higher social support (51, 52, 88), higher social acknowledgment as a victim or survivor (54–56), higher psychological flexibility (47, 89–91), and higher satisfaction with life (43, 44, 52). Interestingly, post-traumatic growth was significantly associated with neither PTSD symptoms nor their severity.

Studies show that younger age and higher extents of social support and SA are associated with higher scores of post-traumatic growth (92). Furthermore, social support was the best predictor for post-traumatic growth in a military sample (93). The current sample was middle-aged and reported a relatively low extent of SA. Furthermore, post-traumatic growth requires a traumatic event that is upsetting enough to cause a subsequent meaning-making of the event by the survivor (94). It is possible that this meaning-making process is absent in the current sample given the demographic variables, as well as the relatively low manifestation of social support and SA as a victim or survivor. This is also reflected by the relatively low manifestation of post-traumatic growth in the whole sample and subsamples. Thus, it is possible that the variability of post-traumatic growth was not pronounced enough to reveal significant associations.

In the next step, group differences between GAF service members with and without PTSD were investigated. The results of these analyses underpinned those of the correlation analyses, with the PTSD group showing significantly higher mean scores in all measures of psychopathology and significantly lower mean scores in all measures of resilience than the non-PTSD, except for post-traumatic growth. The PTSD and non-PTSD groups did not significantly differ in the mean score of post-traumatic growth. This analysis revealed that both groups had relatively low manifestations of post-traumatic growth.

Finally, a mediation analysis with multiple mediators was performed to analyze whether ER is associated with PTSD and whether MI, AA, and SA would mediate the direct path in parallel. The first step identified that difficulties in ER were significantly associated with the severity of PTSD symptoms. After entering the mediators into the model, the relationship between ER and PTSD was partially mediated by SA and AA, but not by MI. The mediating effect of experimental avoidance is in line with previous findings, thus identifying it as an important target for therapeutic interventions and its potential closeness to ER (27, 95).

Of special interest is the mediating effect of SA because it is in line with previous findings in civilians and service members of other nations but conflicts with the findings of a longitudinal study on GAF service members deployed to Afghanistan within the ISAF mission (96). In this report, SA was shown not to have any effect on the occurrence of PTSD. Thus, the role of SA in GAF service members may be hidden in a mediation but still present. Additionally, the relationship between the mediators can be further investigated in this population, which would allow deeper insights since one previous longitudinal study found that experimental avoidance measured by the AAQ-II was a mediator between PTSD symptoms and social support (27).

IThe lack of mediation by MI might be explained by recent study results showing that MI and PTSD are two different pathologies that often occur together (28). MI and PTSD seem to differ in their underlying neurobiology (97). Additionally, MI appears definitely not to be fear-based in comparison to PTSD with different underlying theories (95, 97, 98). Research shows that difficulties in ER are generally associated with psychopathology (18, 24, 99–102). These results are in line with other studies on difficulties with ER in veteran samples. For instance, avoidance as a dysfunctional ER strategy was more often presented by veterans with PTSD than those without it (86). Veterans of operations Iraqi Freedom, Enduring Freedom, and New Dawn who were suffering from PTSD showed more use of expressive suppression and more difficulties with ER than veterans without PTSD (87). Furthermore, psychotherapeutic interventions focusing on ER in veterans were shown to be effective in reducing PTSD symptoms (103), and difficulties in ER were found to be a predictor of PTSD in veterans (17). Thus, the current results suggest that ER is also an important factor for further research and treatments of PTSD in GAF service members.

### Implications

Considering the limitations of this study, the results should be interpreted with caution. However, keeping in mind the limited basis of research on GAF service members, the present results could be seen as an impetus for further research on the relationship between ER and PTSD. The demonstrated mediation of SA and AA allows for further hypothesis-driven research on the population of GAF soldiers. In particular, the role of MI in PTSD has to be investigated to determine whether it is a part of PTSD or whether both are distinct constructs. One recommended approach would be to assess all four constructs that were the focus of this study in further research to provide a broader basis of data.I

### Limitations and Strengths

Several limitations of this study should be noted. First of all, the sample was relatively small, so it is possible that some results remained insignificant due to low power. Nonetheless, for testing mediation, the sample size ensured adequate power using the bootstrapping approach (104). Moreover, sum scores of the construct measurements were used due to the small sample size. Future studies should focus on subscales of measures, especially for ER, SA, AA, and MI. Finally, the sample comprised only males, so the results cannot be generalized to female GAF service members. Generally, the theory-driven approach of the mediation was necessary to check whether the idea of mediation is compatible with our data, but it does not necessarily mean that there is an actual mediation (105).

Nevertheless, the study also has some strengths. Constructs that have repeatedly been reported as having high interest for GAF service members were assessed and investigated in a mediation analysis. The theory-driven choice of constructs also enabled the assessment of a wide range of potential constructs that are associated with PTSD symptoms and their severity among GAF service members, thus leading to solid hypotheses. Finally, the examination of the symptoms and their severity was based on structured diagnostic interview data, whereas the PTSD diagnosis and symptom severity in other studies have often been based on self-rated questionnaires.

## Conclusion

The results of the present study showed that difficulties in ER are associated with the severity of PTSD symptoms in GAF service members. This association is mediated by SA and AA, but not by MI. Thus, future studies should investigate these potentially crucial factors, including measures’ subscales, for better understanding of the development and maintenance of PTSD in GAF service members after a deployment.

Additionally, the role of MI as an individual construct in the association with PTSD should be further investigated in this population. The mediating effect between SA as a victim or survivor on the association of ER and PTSD is promising and requires further studies, especially for the population of GAF service members. The mediating effect of AA on the relationship between ER and PTSD is of special interest since it directly relates to already applied forms of therapy. Studies investigating an applicable use of therapy adaptions covering this effect are greatly encouraged.

## Data Availability Statement

The data of this study are available on request from the corresponding author.

## Ethics Statement

The studies involving human participants were reviewed and approved by Ethics committee of Freie Universität Berlin (85/2014) after internal approval by the German Federal Ministry of Defense. The patients/participants provided their written informed consent to participate in this study.

## Author Contributions

HR, GW, and CK contributed to the conception and design of the study. JS, HR, GW, SS, HN, AK, BM, DW, and SE collected the data. JS and JC performed the statistical analysis. JS, JC, HR, and KK wrote the manuscript. All authors contributed to the article and approved the submitted revision.

## Funding

The German Federal Ministry of Defense (Bundesministerium der Verteidigung) provided government funding for the study. The foundation had no influence on the study design, the collection, analysis, and interpretation of data, the writing of the report, or the decision to submit the manuscript for publication. The Foundation of German Business provided doctoral funding for SE without having influence on the study in any way.

## Conflict of Interest

The authors declare that the research was conducted in the absence of any commercial or financial relationships that could be construed as a potential conflict of interest.
